# Formation of pre-pore complexes of pneumolysin is accompanied by a decrease in short-range order of lipid molecules throughout vesicle bilayers

**DOI:** 10.1038/s41598-020-60348-0

**Published:** 2020-03-12

**Authors:** Bayan H. A. Faraj, Liam Collard, Rachel Cliffe, Leanne A. Blount, Rana Lonnen, Russell Wallis, Peter W. Andrew, Andrew J. Hudson

**Affiliations:** 1 Department of Respiratory Sciences, University of Leicester, Leicester, LE1 9HN United Kingdom; 2 School of Chemistry, University of Leicester, Leicester, LE1 7RH United Kingdom; 3 School of Mathematics, University of Leicester, Leicester, LE1 7RH United Kingdom; 4 Novartis Pharma AG, Basel, Switzerland; 5 Leicester Institute of Structural and Chemical Biology, University of Leicester, Leicester, LE1 7RH United Kingdom

**Keywords:** Membrane biophysics, Chemistry

## Abstract

Oligomers of pneumolysin form transmembrane channels in cholesterol-containing lipid bilayers. The mechanism of pore formation involves a multistage process in which the protein, at first, assembles into a ring-shaped complex on the outer-bilayer leaflet. In a subsequent step, the complex inserts into the membrane. Contrary to most investigations of pore formation that have focussed on protein changes, we have deduced how the lipid-packing order is altered in different stages of the pore-forming mechanism. An optical tweezing apparatus was used, in combination with microfluidics, to isolate large-unilamellar vesicles and control exposure of the bilayer to pneumolysin. By monitoring Raman-scattered light from a single-trapped liposome, the effect of the protein on short-range order and rotational diffusion of lipids could be inferred from changes in the envelope of the C–H stretch. A significant change in the lipid-packing order takes place during assembly of pre-pore oligomers. We were not able to detect a change in the lipid-packing order during the initial stage of protein binding, or any further change during the insertion of oligomers. Pre-pore complexes induce a transformation in which a bilayer, resembling a liquid-ordered phase is changed into a bilayer resembling a fluid-liquid-disordered phase surrounding ordered microdomains enriched in cholesterol and protein complexes.

## Introduction

Cholesterol-dependent cytolysins (CDCs) are a family of more than 20 cytolytic proteins that are highly conserved. A representative member of this family is pneumolysin (PLY), a major virulence factor of the bacterium *Streptococcus pneumoniae*^1^, which is responsible for a wide range of infectious diseases, including pneumonia, bronchitis, meningitis, sepsis and otitis media^2^. These cytolysins, along with the membrane attack complex of complement and perforin^3^, are believed to share a common mechanism for pore formation in lipid bilayers. Structural data have been obtained by cryo-electron microscopy (cryo-EM) showing that oligomeric complexes of PLY, with up to 44 subunits^4^, form transmembrane channels with a diameter of 26 nm^5^. This prior work has also confirmed the multistage nature of the interaction between CDC subunits, in which the self-assembly of a pre-pore complex on the outer-bilayer leaflet takes place before a concerted change in the conformational structure of monomer subunits that leads to insertion into membranes. In more recent work, both cryo-EM and real-time atomic-force microscopy have been used to visualise individual steps in the assembly pathway of a related CDC, suilysin^6^.

PLY, and other CDCs, have four domains, a membrane attachment domain 4 (D4) and three noncontiguous domains, D1–D3. The soluble form of PLY commonly exists as monomers^7,8^. Large circular structures form at higher concentrations^8,9^ and X-ray crystallography has shown that PLY forms linear oligomers in the solid state^9^. Domain 4 is the membrane binding domain and the interaction enables a tryptophan loop at the base of the toxin to penetrate the outer-bilayer leaflet^10–12^. Spectroscopic studies have shown that the environment of certain tryptophan residues in D4 is altered following binding to lipid membranes^13^. Molecular dynamics simulations have demonstrated that PLY adheres, via D4, to the outer bilayer leaflet of phosphocholine membranes, with or without cholesterol, however, in the absence of cholesterol, fewer molecules of PLY dock to the membrane surface and others can dissociate reversibly (in the presence of 30% cholesterol, PLY binding is irreversible)^14^. The same simulations have shown that the residues Trp433, Trp435 & Trp436 (the “tryptophan loop” in D4) and Thr459, Leu460 & Tyr461 (also in D4) interact directly with cholesterol molecules at high concentrations, where Try435 and Trp436 anchor PLY to the lipid bilayer. Changes in the conformation of the tryptophan-rich loop appear to promote monomer-monomer interactions via predominantly hydrophobic interactions mediated by residues on D1, D2 and D3^15^. These contacts result in formation of pre-pore complexes. The assembly process has been shown to be non-cooperative, terminating before the complex inserts into the lipid bilayer^16^.

During pore formation, concerted folding of D2 on each of the monomeric subunits of the pre-pore complex directs D1 and D3 toward the lipid bilayer. A vertical collapse of the pre-pore structure is accompanied by an expansion in ring diameter due to a large change in subunit structure and packing caused by conformation changes within D3 from α-helices to β-hairpins that extend through the lipid bilayer^17^. Salt bridges between the β-hairpins on adjacent subunits stabilise the inserted complex^18^. Time lapse atomic-force microscopy has suggested that three irreversible mechanistic steps exist, with a membrane-attached ring oligomer transitioning to an early pre-pore (with 1 nm drop in height), then to a late pre-pore (with a further 3 nm drop in height), and finally to the transmembrane pore^19^. While the protein-lined inner surface of the pore is hydrophilic, the outside surface of the β-barrel interfacing with the membrane is apolar with large side chains immersed in the lipid bilayer^19^. Most investigations of CDC pores have concentrated on the protein and its conformational changes during pore formation. Thus the pore structures reported in the literature describe a protein-lined hydrophilic channel, with little information being available on the role of, and impact on, lipid conformation during pore formation. This is somewhat surprising given that the propensity for pore formation by CDCs varies between different cells (for example, epithelial cells of the brain are more sensitive than those of the respiratory tract^20^, which points to the lipid composition and structure of membranes being a significant determinant.

An interaction of a specific chemical nature between CDCs and cholesterol is widely assumed to exist and play a critical role in protein binding to membranes^12^, but there is little further understanding about how the packing of lipid molecules affects, and is affected by, the different stages of the pore-formation mechanism. There is evidence that the protein can stabilise the configuration of lipid molecules in which the inner and outer leaflets of the lipid bilayer fuse into a toroidal structure to form proteolipic pores^21^. In addition to ring-shaped oligomers, cryo-EM has also revealed pre-pore and pore forms of arc-shaped oligomers. The pre-pore to pore transition of arc-shaped oligomers is a cooperative process that is more likely to occur at low protein concentration when the assembly process is kinetically arrested^16^. Sub-tomogram classification has been able to detect, in some cases, the absence of lipid within the curve of arc-shaped oligomers^22^.

In this work we sought to address the lack of data on the interaction of lipids with CDCs, using a combination of optical tweezers, microfluidics and Raman spectroscopy to give new insights into the physical changes in a liposomal bilayer exposed to PLY. By extending the study to include various mutants of the protein, with reduced haemolytic activity, we have been able to determine the physical changes taking place during the individual steps of protein binding, the formation of pre-pore oligomers, and the insertion of ring and arc complexes.

## Results and Discussion

### Summary of the experiments

**Figure 1:**
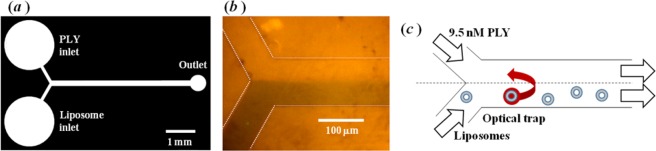
(**a**) The photomask used to fabricate the master template for preparing replicas of a microfluidic component. The two inlet channels of 100 μm-width converge, at a Y-shaped junction, into a channel of 200 μm-width. (**b**) Image of the laminar flow of solutions in the microfluidic device with a dye added to the solution injected at the lower inlet. (**c**) Illustration of the experimental method – A single liposome was optically trapped in the original liposome suspension and then transferred across the laminar flow boundary into the PLY solution.

In this study, a microfluidic approach was used to deliver liposomes and protein to optical tweezers. The microfluidic components were similar in design to those reported previously by Ramser and co-workers^23^, and fabricated by soft lithography (see Methods; an illustration of the photomask used for preparing the master template is shown in Fig. 1(a)) Co-flowing streams of a dilute liposome suspension, and a protein (PLY) solution, remain separated in the channels due to laminar flow conditions. Diffusional mixing of the reagents was insignificant in the flow upstream of the Y-shaped junction for a short distance. Visualisation of the laminar-flow boundary was made by replacing one of the injected fluids with a solution containing a dye (see 1(b)), and an illustration of a typical experiment is shown in 1(c).

A single liposome from the flow of a liposome suspension was optically trapped downstream of the Y-shaped junction, and subsequently transferred across the laminar flow boundary into the PLY solution. This was achieved by affixing the microfluidic component to the kinematic stage on the optical-tweezing apparatus, and then moving the stage (without altering the optical alignment of the trap) in order to drag the trapped liposome between the separated fluid flows. The kinematic velocity of the fluid downstream of the junction was 80 μm s^−1^. In the experiments, the liposomes were initially trapped at a distance of <100 μm downstream of the Y-shaped junction. An estimate of the diffusion coefficient of PLY is ~100 μm^2^ s^−1^, which indicates that the protein is likely to have been present in the flow of the liposome suspension at ~15 μm from the centreline of the microfluidic channel. Therefore, liposomes were initially trapped near one edge, and translated close to the opposite edge, of the 200 μm-wide microfluidic channel. Whilst the liposome was held by the optical tweezers in the solution containing the bacterial toxin, the C–H stretching band was monitored continuously by Raman microspectroscopy. The temperature of the microscope stage was maintained at 37 °C.

**Figure 2:**
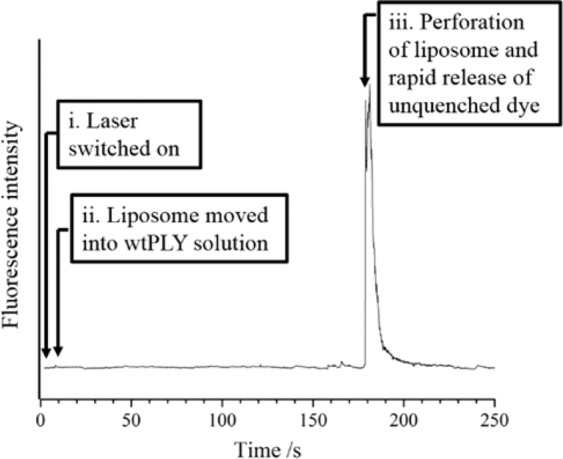
Fluorescence measurement of calcein leakage from an optically-trapped liposome isolated in a flow of PLY (0.1 μM). The quantum yield for fluorescence emission from calcein molecules within the intact liposome is low due to a self-quenching mechanism at [calcein]_0_ = 70 mM. Leakage of calcein from the liposomal bilayer, following exposure to PLY, is observed after an interval of approx. 180 s. All the calcein molecules exited the liposome, and diffused away from the waist of a 488 nm laser, after a further 10 s.

Interaction of wild-type PLY with an optically-trapped liposome is demonstrated by the fluorescence measurement shown in Fig. 2. For this example, the liposomes (1 part POPC: 1 part cholesterol (chol)) were prepared in a hydrating solution containing 70 mM calcein. At this concentration, self quenching leads to a low quantum yield for fluorescence emission from calcein. The leakage of calcein from an optically-trapped liposome exposed to PLY was monitored using the optical system for Raman microspectroscopy. In this case, the leakage of incorporated dye gives rise to a high quantum yield of fluorescence (with exposure to 488 nm light). In order to monitor fluorescence, the 488 nm laser power was attenuated to 3 μW (in the plane of the optical trap) to avoid saturation of the detector with intense signals; the fluorescence emission (>505 nm) was steered directly onto a CCD (bypassing the grating in the spectrograph); and spectra were collected in 1 s intervals with an integration time of 10 ms. A high concentration of PLY was used in this experiment (0.1 μM; a lower concentration was used in all subsequent experiments). The integrated intensity of the fluorescence emission is shown in Fig. 2 as a function of time. The optically-trapped liposome was initially located in the flow of the diluted suspension. At *t* ≈ 5 s, the liposome was moved across the laminar flow boundary into the solution containing PLY. Leakage of calcein from the interior of the vesicle, across the liposomal bilayer, was first observed after *t* ≈ 180 s. A transient fluorescence signal was observed from the onset of perforation of the bilayer for an interval of time until all the incorporated calcein molecules had exited the liposome, and diffused sufficiently far from the waist of the 488 nm laser. The time taken for the contents of the perforated liposome to exchange with the buffer solution in the microfluidic flow was ~10 s. After this time, no further fluorescence signal was detected.

### Electron microscopy (EM) images of oligomers of PLY and mutants in synthetic lipid bilayers

The interface between monomer sub-units in PLY is created by the concave face of one monomer packing against the convex face of a neighbour, leading to exclusion of water^15^. A total of 85 residues are located at the interface, and these residues are well conserved in other members of the cholesterol-dependent cytolysins. In our previous work, we created a series of mutations that were designed to interfere with protein oligomerisation by removing the shape or charge complementarity between surfaces on adjacent proteins. Of these, D205R and N339R, with mutations in domain 1 (D1), lost the ability to lyse sheep erythrocytes, whereas T304R, with the mutation in domain 3 (D3), had a dramatically lower activity (1/300) compared to wild-type PLY^15^.

**Figure 3:**
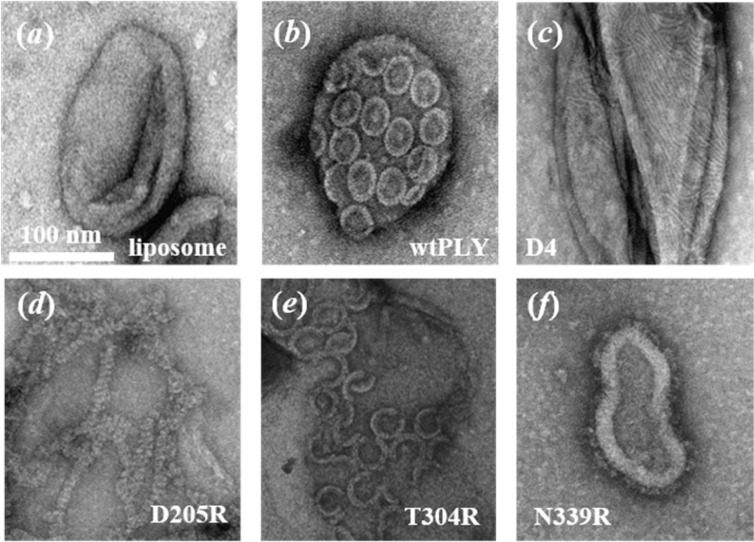
Transmission electron micrographs of POPC/chol liposomes (1:1 mol ratio) incubated (**a**) in the absence of protein, and in the presence of (**b**) wild type (wt) PLY, (**c**) the truncated mutant comprising domain 4 (D4) only, (**d**) D205R, (**e**) T304R and (**f**) N339R.

To determine the effects of the mutations on pore formation, samples of pure POPC/chol liposomes (Fig. 3(a)), and vesicles that were incubated with PLY, a truncated mutant comprising domain 4 (D4) only, and point mutants D205R, T304R and N339R (3(b–f)) were investigated by scanning electron microscopy. As before^24^, wild-type PLY was found to form complete oligomeric rings covering the majority of the bilayer surface (3(b)). Discrete oligomeric structures were not observed in EM images of the truncated mutant, consisting of D4 only (3(c)). In this example, the stripped pattern could be the result of linear assemblies of protein in the lipid bilayer.

The inactive point mutant, PLY D205R, still oligomerised on liposomal bilayers. However, unlike the wild-type protein, it appeared to form long linear chains rather than rings or arcs (3(d)). This mutation, therefore, does not prevent oligomerisation but instead prevents oligomers from circularising to produce rings. By contrast, the other haemolytically inactive mutant, PLY N339R, did not oligomerise on unilamellar lipid vesicles (see 3(f)). Instead, the monomer units were distributed across the surface of the lipid bilayer. Thus although both mutants are functionally inactive, they have different defects. PLY D205R retains its ability to oligomerise on the membrane but cannot form pores, while PLY N339R completely fails to oligomerise on lipid bilayers. Interestingly, the T304R mutant, with reduced haemolytic activity, had an interemediate phenotype, forming many arc structures in EM images (3(e)). An explanation for the lower haemolytic activity of T304R is that the mutation in the protein structure interferes with either the association of PLY monomers or the concerted refolding of D2 in PLY oligomers (since arc oligomers are known to insert in lipid bilayers^22^).

### Measurement of pore formation by PLY and mutants in synthetic lipid bilayers

The haemolytic activities of the truncated mutant, D4, and the point mutants D205R, T304R and N339R have been reported before^15^. To compare their activities in synthetic bilayers, liposomes (1:1 POPC/chol) were filled with a molecular dye, calcein, at a concentration above the threshold for self-quenching of fluorescence. Leakage of calcein was monitored from liposomes incubated with various concentrations of PLY, D4, D205R and N339R. The results are shown in Fig. S1. The formation of transmembrane pores in liposomal bilayers by PLY led to an increase in fluorescence via a 1^st^-order process (S2). There was no significant increase in fluorescence observed following addition of the point mutants, D205R and N339R, to liposomes in concentrations up to 20 nM, confirming that they do not form functional pores.

### The effect of wild-type PLY on the degree of membrane order and the rotational diffusion of lipid molecules

A Y-shaped microfluidic chamber was employed to transfer a single liposome, held by optical tweezers, from a suspension of vesicles into a solution containing PLY; see Fig. 1(c). Laminar flow conditions, and slow diffusion of protein between the co-flowing streams, ensured an instantaneous exposure of the isolated liposome to 9.5 nM PLY. The lipid packing order in the membrane bilayer was monitored in real time by recording the C–H stretching region of the Raman spectrum.

**Figure 4:**
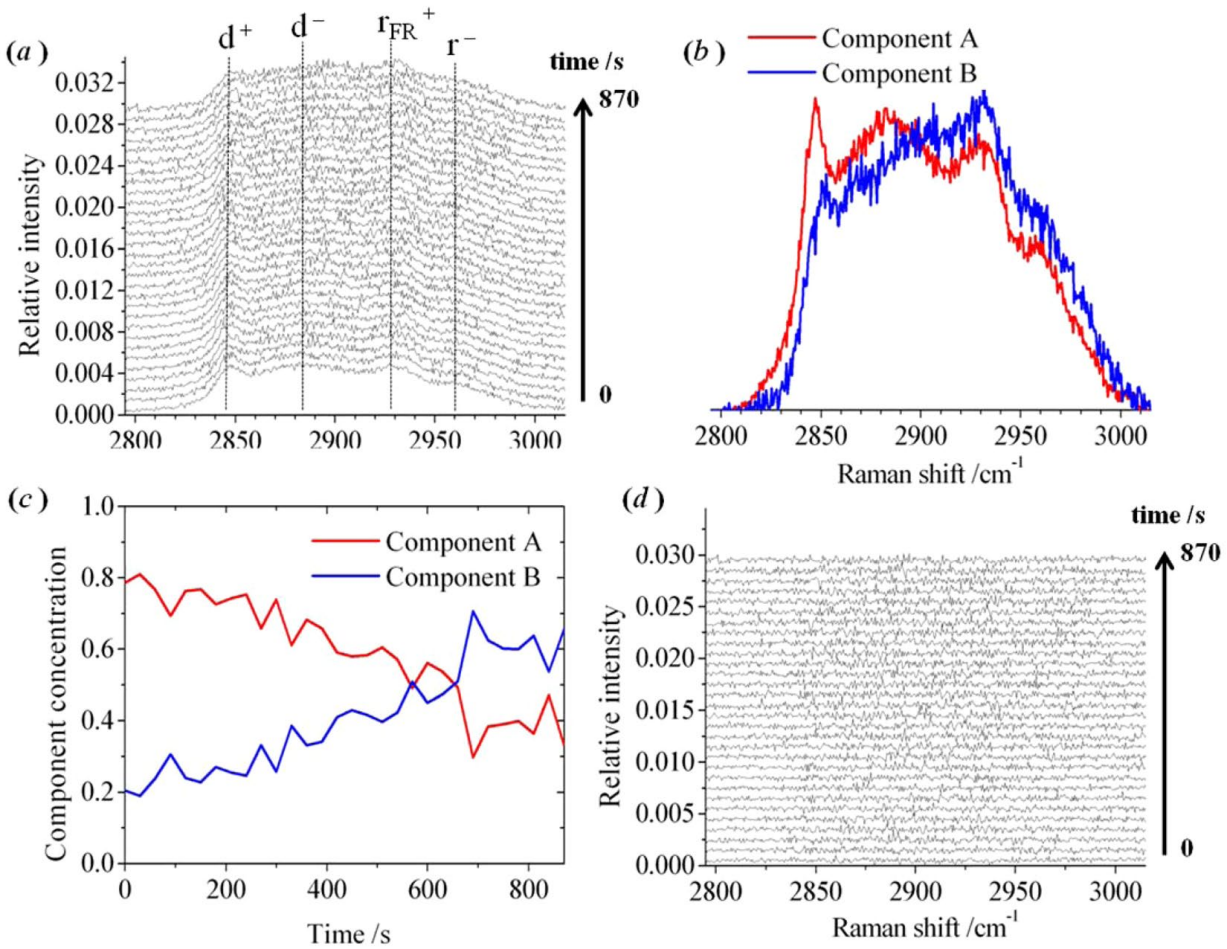
Raman spectra of an individual optically-trapped POPC/Chol liposome, in the region of the C–H stretching band. (**a**) A sequence of experimental Raman spectra was recorded at 30 s intervals. The overall duration of the experiment was 29.5 minutes following the movement of the liposome into the PLY solution. (**b**,**c**) The pure spectral profiles and concentration profiles, respectively, for two components obtained by multivariate curve resolution. (**d**) The residuals for the Raman spectra following optimisation of the component profiles.

An example of a sequence of Raman spectra measured from an optically-trapped liposome (1:1 POPC/chol) exposed to PLY is shown in Fig. 4(a). The initial spectrum was recorded immediately after the liposome was captured from the vesicle suspension and prior to exposure to PLY in the adjacent laminar flow. The final spectrum was recorded at 870 s in the laminar flow containing 9.5 nM PLY. The C–H region comprises the symmetric (d^+^; 2840 cm^−1^) and antisymmetric (d^−^; 2870 cm^−1^) methylene stretch, the Fermi resonance of the symmetric methyl stretch (r_FR_^+^; 2920 cm^−1^) and the antisymmetric methyl stretch (r^−^; 2960 cm^−1^)^25^.

The sequence of Raman spectra was analysed using a multivariate-curve resolution (MCR) alternating-least squares (ALS) algorithm^26,27^. This is a chemometric approach for extracting the spectral and concentrations profiles of the underlying components in large sets of recorded spectra. The fitting of two components to the sequence of Raman spectra in Fig. 4(a) accounted for >99% of the variance in the experimental data. Further detail on the application of MCR-ALS to analyse the C–H stretching band in the Raman spectra of lipid vesicles is given in our earlier publication^28^. The spectral profiles for the pure components, A and B, are shown in 4(b). During the course of the experiment, the relative weight of component A decreased smoothly, accompanied by an increase in the weight of component B, as shown in 4(c). In our previous study^28^, MCR-ALS was applied to a series of Raman spectra from an optically-trapped vesicle of the same size and lipid composition recorded between 20 and 60 °C (*i.e*. in the absence of protein). The existence of a pair or pure components was sufficient to account for the temperature-dependent spectral profile, and was attributed to regions of the lipid bilayer with different short-range order. The pure component spectra reported in that publication^28^ bear a close similarity to the pair shown in 4(b) and, therefore, the changes exhibited by the lipid bilayer following exposure to PLY are equivalent to those following heating to 60 °C. The ratio of concentrations of the pure components was found to be 0.95:0.05 in a spectrum measured at 20 °C, and this gradually changed to 0.00:1.00 in a sequence of spectra measured at intervals up to 60 °C^28^. For the example shown in Fig. 4, the temperature of the optically-trapped liposome was fixed at an intermediate value of 37 °C and, as a consequence, the initial spectrum contains a mixture of the pure components with a ratio of concentrations of 0.80:0.20. During the course of the experiment, the exposure of the liposome to PLY led to a reduction in the ratio of the concentrations of pure components to 0.35:0.65 within the membrane. The residuals for the analytical fit to the raw experimental data are shown in 4(d). [Relative concentrations are given to ±0.05.]

Replicates of the experiment were performed using point mutants of PLY with a wide range of different haemolytic activities (HAs) relative to wild-type PLY, namely, R226A (HA 0.06^29^), T88E (0.04^29^), W433F (0.01^30^), V341R (~1^29^), K268A (0.4^29^) and L11R (0.7^29^). The method for measuring relative haemolytic activity has been described earlier^29^. All of these point mutants exhibited the same pattern in time-dependent measurements of Raman spectra for POPC/chol liposomes (see S8 and S9). Thus the differences in lipid-lipid interactions before and after exposure to PLY are independent of hemolysis. A control experiment performed in the absence of protein is shown in our previous publication^28^.

In our previous study^28^, the temperature-induced change in the appearance of the C–H stretching band of an optically-trapped lipid vesicle was interpreted to correspond to a gradual decrease in the lateral packing order of hydrocarbon chains and increase in rotational diffusion of lipid molecules in the fluid phase of POPC/chol bilayers. We judged that the same interpretation can account for the time dependence shown by the spectral profiles in Fig. 4 for a POPC/chol liposome exposed to PLY at a fixed temperature of 37 °C. This is the first time that an observation has been made on how PLY changes the physical properties of the lipid bilayer.

In the absence of protein in lipid bilayers, the intercalation of cholesterol with phosphocholine molecules will promote *cis*-to-*trans* conformational changes of the hydrocarbon chains in a fluid-lipid bilayer. This results in a higher degree of short-range order relative to pure phosphocholine bilayers^31^. The temperature-dependent behaviour of POPC/chol bilayers has been inferred, by others^32^, from measurements of membrane fluidity via steady-state fluorescence anisotropy measurements of a membrane dye. At room temperature, a binary (1:1) mixture of POPC and cholesterol was proposed to exist in a liquid-ordered phase, L_o_, sharing characteristics of the gel (L_β′_) and fluid (liquid-disordered, L_d_) phases. At higher temperatures, the L_o_ and L_d_ phases were proposed to co-exist in POPC/chol bilayers, however, while consistent with studies of membrane fluidity, the existence of a thermotropic phase transition, L_o_ → L_o_/L_d_, cannot be confirmed by differential scanning calorimetry^28^. There is debate in the literature^32,33^ as to whether the properties of binary mixtures (such as POPC/chol) should be described by phase-separated L_o_ and L_d_ regimes, or by gradual changes in a largely homogeneous lipid bilayer. An alternative model to account for heterogeneity in lipid bilayers is the dynamic formation and dissolution of ordered nanostructures (lipid rafts), which can increase in number, and be kinetically-arrested, by protein embedded in the bilayer^33^. Lipid rafts are assumed to resemble L_o_ structures (enriched in both cholesterol and protein) floating in a fluid-L_d_ membrane. The clear similarity of the results shown in Fig. 4 to the observed changes in the profile of the C–H stretching band on heating a POPC/chol liposome up to 60 °C^28^ points to the conclusion that the lipid bilayer is converted from a state resembling a pure L_o_ phase into a state resembling co-existing L_o_/L_d_ phases in the presence of wild-type PLY at 37 °C. An assignment of the pure components, derived from the sequence of Raman spectra, can be made to lipid molecules in an ordered environment (L_o_; Component A) and in a disordered environment (L_d_; Component B), respectively. Thus, in the presence of PLY, the environment of lipid molecules becomes more disordered. These assignments are appropriate for the data in Fig. 4 obtained by exposing a liposome to PLY at 37 °C, and previous data^28^ obtained by heating a liposome between 20 and 60 °C.

**Figure 5:**
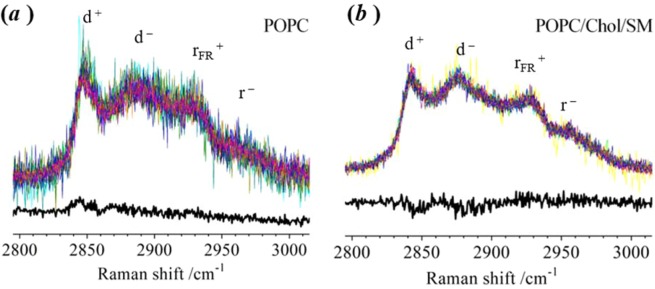
Comparison of Raman spectra for an optically-trapped liposome in the absence and presence of wild-type PLY. (TOP) In the presence of PLY, the individual spectra recorded from different liposomes are superimposed. (BOTTOM) Subtraction of the average spectrum recorded from 10 individual liposomes in the absence of PLY from the average spectrum recorded in the presence of PLY gives the difference spectrum. No significant features remain in the difference spectrum. Liposomes were prepared (**a**) from pure POPC; and (**b**) from a ternary mixture (POPC/chol/SM) with a mole ratio of 1:1:1. There are 16 replicates included in both (**a**,**b**) for liposomes in the presence of PLY.

Similar measurements have been made using liposomes comprising pure-POPC and a ternary mixture of POPC, cholesterol and sphingomyelin (SM). Wild type PLY is not expected to bind to a lipid bilayer composed entirely of POPC and, as expected, the C–H stretching band was found to be identical in the spectra recorded from liposomes in the presence or absence of protein (Fig. 5(a)). A ternary mixture of POPC/chol/SM lipids, in mol ratios of 1:1:1 or 1:0.5:0.5, is expected to have a non-homogeneous distribution and has been proposed to exhibit co-existing L_o_ and L_d_ phases in bilayers at 37 °C^32^. The 1:1:1 composition of lipids is widely used as a system for observation of lipid rafts. We have been unable to detect any difference in the lateral packing order of hydrocarbon chains or rotational diffusion of lipid molecules in the presence and absence of PLY for POPC/chol/SM bilayers with a 1:1:1 mol ratio (see 5(b)) and a 1:0.5:0.5 mol ratio (see S3). We speculate that the induction of disordering in the bilayer by PLY is more difficult to see against the background level of disorder of the 1:1:1 POPC/chol/SM bilayers. Thus, the PLY oligomer should still cause a change in lipid distribution and membrane order but this falls below the limit of detection for the Raman measurement. Prior to the presence of any protein, 1:1 POPC/chol bilayers exist in a single phase corresponding to L_o_^32^. We speculate that the reason a substantial change takes place to produce a L_d_ phase co-existing with a L_o_ state is that the L_d_ phase facilitates transport of PLY monomers, whereas the L_o_ phase facilitates assembly of PLY oligomers. If this is the case, then the phase transition (L_o_ → L_o_/L_d_) must take place following the initial formation of PLY oligomers to promote the assembly of larger structures capable of insertion into the bilayer. The entire absence of a L_d_ phase, prior to PLY addition, in 1:1 POPC/chol bilayers means that there is a greater change in lipid order during the experiment and this is easily detected by the Raman measurement. Subsequent experiments have utilised the 1:1 POPC/chol bilayers exclusively in order to compare the outcomes of measurements using the truncated mutant, point mutants and locked mutants of PLY.

### Investigating the different steps leading to the assembly of PLY to form transmembrane pores in lipid bilayers

The purpose of the next series of experiments was to determine whether the decrease in short-range order and increase in rotational diffusion of lipids is caused by the initial binding of PLY to the lipid bilayer, the formation of oligomeric complexes (i.e. pre-pore forms), or, alternatively, the insertion of protein complexes into lipid bilayers. This was done by studying a collection of PLY mutants of differing haemolytic activities, namely: (i) D4 (HA < 0.001^15^) and PLY N339R (HA < 0.001^15^), both of which bind to lipid bilayers but do not oligomerise (3(c,f)); (ii) PLY D205R (HA < 0.001^15^), which binds and oligomerises on lipid bilayers but assembles into linear oligomers rather than rings (3(d)); and (iii) PLY T304R (HA ~0.003^15^), which forms pre-dominantly incomplete-arc structures in lipid bilayers (3(e)). We have also included data from experiments using two different ‘locked’ mutants. For these mutants, a pair of point mutations was made to the amino acid sequence in D2 of the protein: T55C + V163C in one mutant, and A262C + W278C in the other^34^. These mutants were designed to retain the capability to bind to lipid bilayers and assemble into oligomers but the cross-linking of cysteine residues hinders the conformation change in D2 that must take place prior to insertion of the oligomer into the lipid bilayer.

**Figure 6:**
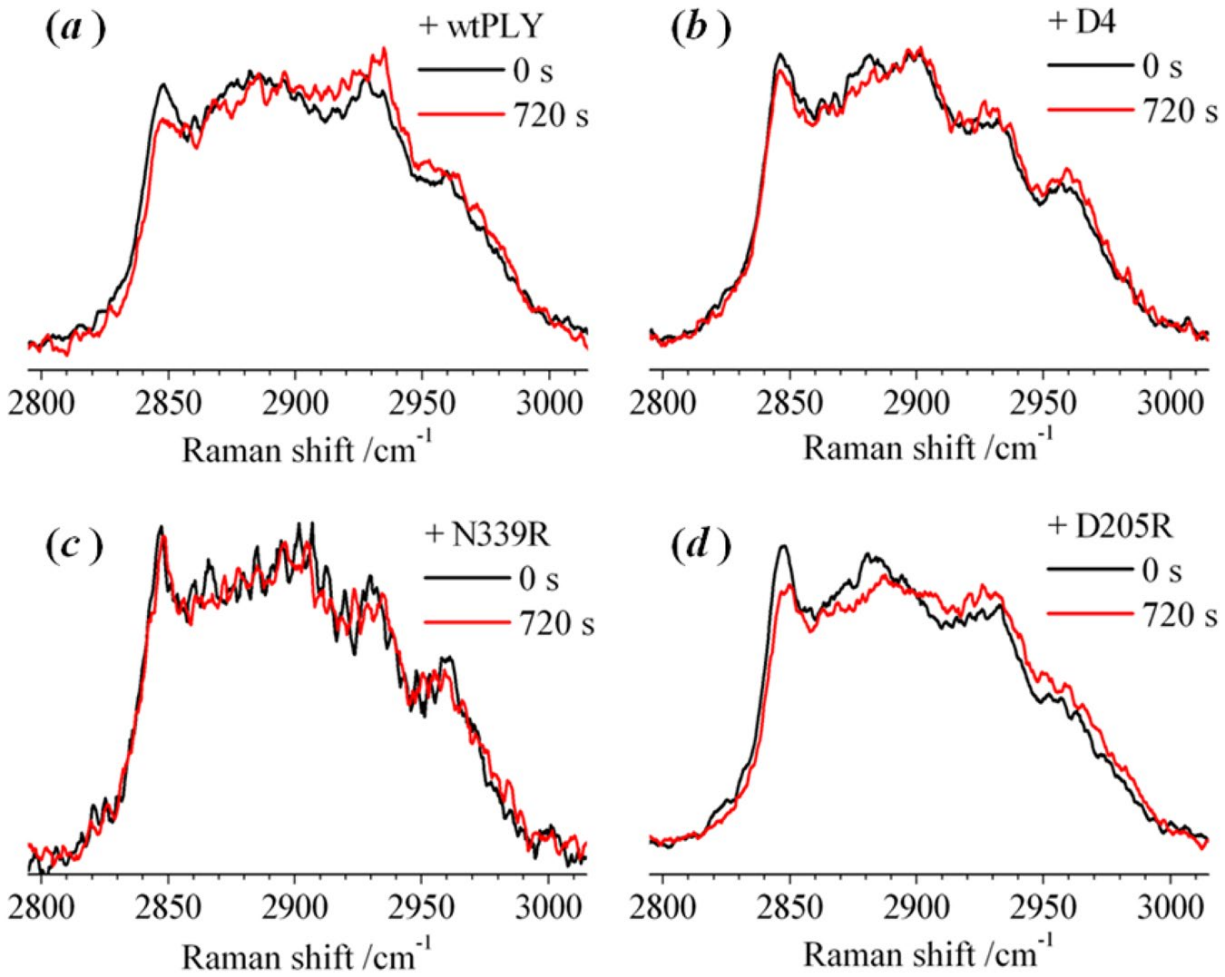
Raman spectra of an individual optically-trapped POPC/Chol liposomes (1:1 mol ratio), in the region of the C–H stretching band, recorded at 30 s intervals following exposure to (**a**) PLY, (**b**) the PLY D4 and the point mutants (**c**) N339R and (**d**) D205R. Only the raw experimental spectra recorded after 0 s and 720 s are shown. Analysis of the full set of spectra for PLY are shown in Fig. 1, for D4 and N339R in the Supporting Information (**S6**), and for D205R in Fig. 7.

The microfluidic approach (described above) was used to monitor the change in the Raman spectrum of an optically-trapped liposome (1:1 POPC/chol) transferred into a solution containing D4, D205R or N339R. Raman spectra were recorded in intervals of 30 s, but only the Raman spectrum recorded initially and after 720 s are shown in Fig. 6; a full presentation of the recorded data is provided in the Supporting Information (S6). The initial and final spectrum, recorded in the presence of PLY, shown in 6(a) is reproduced from the data in 4(a). There is no obvious change in the appearance of the Raman spectrum measured in the presence of either D4 or N339R (see 6(b) and (c), respectively), showing that the structure of the lipid bilayer is not affected by the initial binding of the protein, and that the interaction of the tryptophan loop of the protein with cholesterol in the bilayer has an insignificant overall effect on the membrane order and the rotational diffusion of lipid molecules.

**Figure 7:**
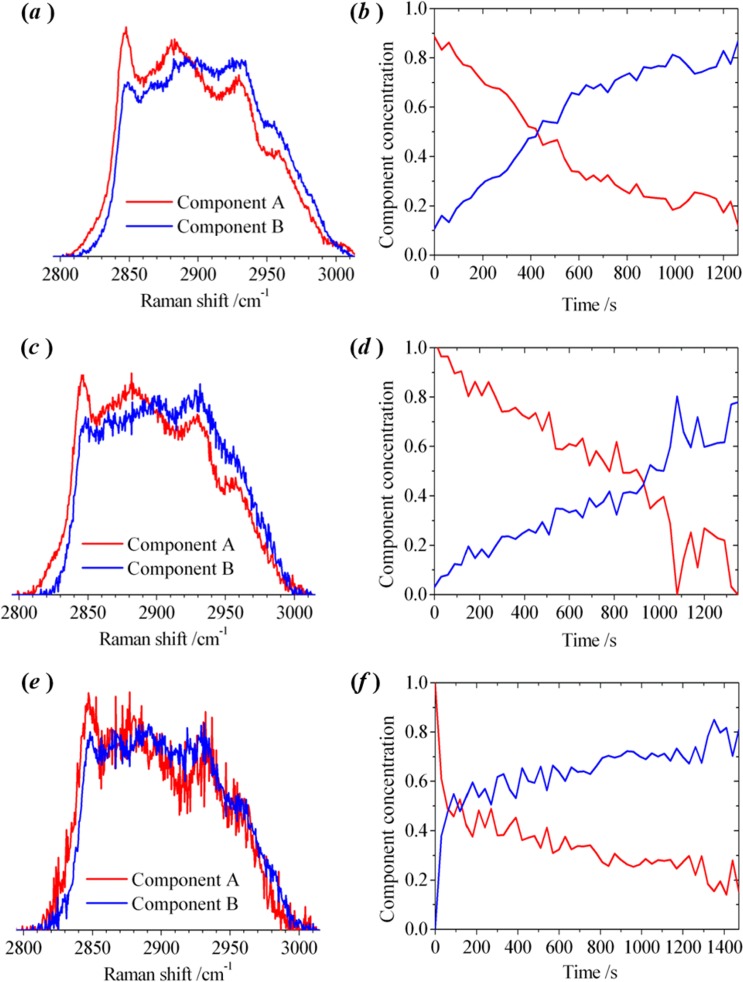
Multivariate-curve resolution (MCR) analysis of Raman spectra recorded from an individual optically-trapped POPC/Cholesterol liposome (1:1 mol ratio), in the region of the C–H stretching band. Sequences of spectra were recorded at 30 s intervals. The pure spectral profiles (left) and the concentration profiles (right) were obtained by MCR analysis for a liposome transferred into a laminar flow of PBS buffer in the presence of the point mutant D205R [(**a**,**b**)] and the locked structures with dual-point mutations T55C + V163C [(**c**,**d**)] and A262C + W278C [(**e**,**f**)]. The full set of raw experimental spectra is shown in the Supporting Information (S7).

Surprisingly, the D205R mutant caused similar changes to the Raman spectrum as wild-type PLY (see **6 (*a*)** and **(*d*)**) despite not forming ring oligomers in lipid bilayers. An analysis of the experimental data for D205R, using the multivariate algorithm, is shown in Fig. 7(a,b). The pure component spectra derived from Raman spectra for D205R are similar to those derived with PLY (c.f. 4(b) and 7(a)). Similar changes were also seen in the Raman spectrum obtained using the locked mutants, T55C + V163C (7(c,d)) and A262C + W278C (7(e,f)), which oligomerise on membranes to form pre-pore structures, as does PLY, but cannot undergo the conformational changes to form the mature pore. These results show that a significant change in the packing of lipid molecules takes place during the assembly of protein sub-units to form pre-pore oligomers. The initial stage of protein binding to the membrane (observed in isolation with D4 and N339R) does not appear to modify the packing of the lipids in a way that was reflected by changes in the C–H stretching band.

Formation of pre-pore complexes induce a transformation in the lipid bilayers containing cholesterol (50 mol%), in which a bilayer, resembling a liquid-ordered (L_o_) phase is changed into a bilayer that resembles a fluid-liquid-disordered, L_d_, phase surrounding L_o_-domains enriched in cholesterol and protein complexes. The contribution of the disordered environment of lipid molecules (L_d_) appears to dominate the Raman spectra measured at the conclusion of the experiments with PLY, D205R and the locked mutants, all of which have the capability to form oligomers in lipid bilayers.

**Figure 8:**
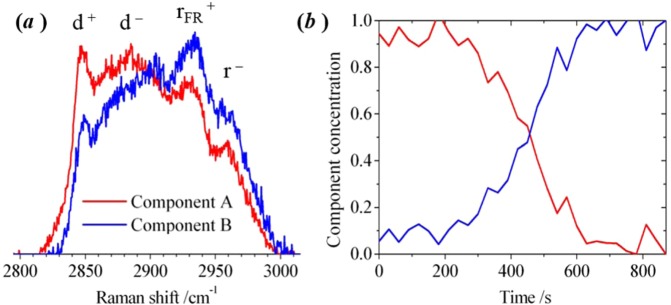
Multivariate-curve resolution (MCR) analysis of Raman spectra recorded from an optically-trapped POPC/Cholesterol liposome (1:1 mol ratio), in the region of the C–H stretching band in the presence of the point mutant T304R. Sequences of spectra were recorded at 30 s intervals. (**a**) The pure spectral profiles and (**b**) the concentration profiles were obtained by MCR analysis for a liposome transferred into a laminar flow of PBS buffer. The full set of raw experimental spectra is shown in the Supporting Information (S7).

The results obtained with liposomes exposed to T304R were unlike any of the other observations. Addition of T304R led to a substantial change in the appearance of the Raman spectrum of a POPC/chol liposome. In the measurements made using T304R, the overall decrease in the peak height of d^+^, and corresponding increase in r_FR_^+^, was larger than that observed with PLY. An example of an experiment, using a microfluidic approach, in which a single liposome (1:1 POPC/chol) was monitored continuously in the presence of T304R is shown in Fig. 8(a,b). The differences between d^+^ and r_FR_^+^ in 8(a) for the pure components derived by MCR-ALS are more noticeable than seen with PLY (4(b)). As seen in 3(c), T304R forms oligomers but predominantly as arcs rather than rings leading to the conclusion that the arcs cause more pronounced change in membrane order and rotational diffusion of lipid molecules than occurs with the ring oligomers. Assuming that the formation of an arc structure leads to a tear in the lipid bilayer (consistent with observations of the absence of lipid molecules in the curve of the arc^22^), then the more pronounced change in membrane order could be attributed to disordered lipid molecules adjacent to the edge of the perforated bilayer that is not stabilised by protein. Molecular dynamics simulations have shown that cholesterol molecules remain associated with D4 of PLY oligomers and that the interfacial region between the membrane and inserted β-sheets are devoid of cholesterol^14^. This is consistent with our conclusions from experimental data that the arc oligomers exert a significant influence on lipid distribution.

Consistent with the conclusion that PLY arcs have a different effect on lipid organisation than the PLY rings, all the point mutants that retain the capability to form ring oligomers (W334F, K268A, T88E, L11R, V341R and R226A) exhibited results from the Raman experiments resembling that obtained using wild type PLY (S8 and S9).

## Summary and Conclusion

We have made the first observation of changes in the physical properties of lipid molecules in areas of lipid bilayer surrounding oligomers of PLY. Addition of pneumolysin at a fixed temperature to a lipid bilayer containing a mixture of a phosphocholine lipid and cholesterol led to a gradual change in the envelope of the C–H stretching band in the Raman spectrum. This change resembled that observed on heating a lipid bilayer of the same composition of lipid molecules (reported in an earlier publication^27^). As a consequence of the similarity between the data in the two experiments, and our understanding of the effect of temperature on membrane bilayers, it can be concluded that there is a decrease in membrane order and an increase in rotational diffusion of lipid molecules following addition of PLY to cholesterol-containing bilayers.

A multivariate analysis indicated that a model consisting of two weighted components was sufficient to account for the variation in the spectral profiles recorded as a function of time following exposure of the lipid bilayer to PLY. A possible assignment of the two components is to lipid-bilayer domains with differing degrees of short-range order: *i.e*. resembling a liquid-ordered phase (L_o_) and a fluid-liquid-disordered phase (L_d_) a L_o_, phase and a fluid-L_d_ phase. The weight of the component with higher order, L_o_ decreases gradually following the exposure of the liposomal bilayer to PLY, with a commensurate increase in the weight of the component with lower order, L_d_. At the conclusion of the sequence of recorded Raman spectra, a predominantly disordered (L_d_) assembly of lipid molecules must surround ordered (L_o_) microdomains enriched in cholesterol and protein complexes.

Our results show how lipid-lipid and lipid-protein interactions change during the self-assembly of protein oligomers. The Raman measurements were sensitive to the changes in lipid packing order that take place during the formation of protein complexes, however, the method was not sensitive to further changes in the membrane structure that occur when a pre-pore complex inserts into the lipid bilayer. By studying point mutants of PLY, truncated mutants, and locked structures containing dual-point mutations, we have deduced that the decrease in membrane order and increase in rotational diffusion in POPC/chol lipid bilayers takes place during the assembly of protein sub-units to form pre-pore oligomers. There is no change in membrane order detected on binding of the protein to the lipid bilayer, and no further change in membrane order detected on insertion of complete oligomer structures into the lipid bilayer.

## Methods

### Lipid vesicles

Lipid vesicles, with a mean diameter of approximately 1 μm, were prepared, as before^28^, from a pure phosphocholine lipid (1-palmitoyl-2-oleoyl-sn-glycero-3-phosphocholine, POPC; Avanti Polar Lipids 850457 (16:0–18:1)), a binary mixture (1:1, 4:1 and 1:2 mol ratio) of POPC and cholesterol (chol; Avanti Polar Lipids 700000 (ovine wool)) and a ternary mixture (1:1:1 mol ratio) of POPC, chol and sphingomyelin (SM; Avanti Polar Lipids 860061 (egg)). A 1:100 dilution of the stock suspension, in phosphate-buffered saline (PBS 1×; pH 7.4), was used in the experiments. For the measurements reported in Figs. 2 and S2, the lipid mixtures were rehydrated in PBS containing 70 mM calcein (Sigma Aldrich C0875). The liposomes were centrifuged at 13,000 g for 30 minutes; the resulting pellet was washed to remove unincorporated dye and then re-suspended in PBS. The production and purification of wild-type, D4 and point mutants of pneumolysin has been described elsewhere^15^. Unless otherwise stated, the liposomes were exposed to 9.5 nM PLY in PBS (1×; pH 7.4).

### Electron microscopy

Wild-type PLY, D4 and selected mutants (D205R, T304R and N339R), were incubated with unilamellar liposomes (1:1 POPC/chol). Mixtures were subsequently stained with 1%w/v uranyl acetate on a copper grid to visualise membrane-bound oligomeric structures by negative-stain transmission-electron microscopy EM.

### Raman microspectroscopy

A bespoke microscope apparatus was used for optical tweezing and the measurement of Raman spectra from trapped unilamellar vesicles. This has been described previously in detail^28^. The optical tweezers are formed by a tightly-focussed continuous-wave laser at 1070 nm. A single liposome could be trapped with a power of 20 mW (estimated power in the plane of the optical trap), and the temperature regulated at 37 °C on a heated stage. Spectroscopic measurements were made by focussing a continuous-wave 488 nm laser (3 mW) onto the optically-trapped particle and collecting the inelastic-scattered light. The power of the 488 nm laser alone was insufficient to optically trap a liposome. A confocal-based detection scheme for Raman-scattered light ensured that sensitive measurements could be made from individually-trapped particles. Spectra were acquired continuously between 2800 and 3200 cm^−1^ using a Czerny-Turner scanning monochromator with an integration time of 30 s. Intensities of scattered light were measured in increments of 0.018 nm (~0.5 cm^−1^). The optical resolution of the spectrograph was 2 cm^−1^, and the precision of a wavenumber measurement was 0.5 cm^−1^. The sequences of Raman spectra were normalised in the C–H stretching region between 2780 and 3030 cm^−1^ prior to analysis using the multivariate algorithm.

### Fabrication of microfluidic devices

A photomask containing negative images of the pattern of microfluidic channels was reproduced at 128,000 dpi on a chrome layer supported on 0.060′′-thick soda lime (JD Photo-Tools Inc.). A single copy of the pattern is shown in Fig. 1(a); the pattern was repeated in multiple columns and rows on the photomask. The width of the fluidic channels into which the liposome dispersion and the protein solution were injected was 100 μm, and these converged at a Y-shaped junction into a fluidic channel of 200 μm-width.

The master template was fabricated by spin coating (500 rpm, 30 s, followed by 1500 rpm, 30 s) a permanent epoxy negative photoresist (SU-8 2050, MicroChem Corp.) onto the polished surface of a 2′′-diameter boron-doped silicon substrate ({100} plane, 280 μm-thickness; MicroChemical GmbH). A uniform photoresist layer of 40 μm thickness was obtained following a soft bake at 95 °C for 1 min. The photomask was placed in contact with the coated silicon wafers and illuminated by uniform UV light at 365 nm with a total energy of 550–650 mJ cm^−2^. Following a post-bake at 95 °C for 6 min, the photoresist was developed in Microposit EC solvent (MicroChem Corp.) and cleaned with isopropyl alcohol. An optical profilometer (Zeta-20, Zeta Instruments Inc.) was used to check the three dimensional surface pattern on the silicon wafers.

Replicas of the pattern, shown in Fig. 1(a), were produced in polydimethylsiloxane (PDMS; approximately 5 mm-thick layer). The outline of a rectangle (approximately 25 by 35 mm) surrounding 12 copies of the microfluidic design was cut into the cured PDMS, and the enclosed segment was separated from the silicon wafer. A biopsy punch, 1.0 mm-internal diameter, was used to bore holes at inlets and outlets on the patterns transferred to the PDMS. The PDMS surface and a #1 cover glass (Menzel-Gläser, 24 mm by 50 mm) were activated with an oxygen plasma for 1 min at 0.1 mbar and 28 W (MiniFlecto-PC-MFC, Gala Instrumente GmbH). The patterned surface of the PDMS was then bonded to the cover glass, and placed in an oven at 65 °C for 1 hour. The connections to inlet and outlet channels were made by inserting 0.042′′-O.D. tubing (Microbore PTFE tubing, Cole-Parmer Ltd.) into the bored holes.

The suspension of liposomes and a 9.5 nM solution of PLY (0.5 μg/ml) were delivered to separate inlets of the microfluidic component by a dual-syringe pump (Harvard Apparatus Ltd.) using a pair of gas-tight glass syringes, with Luar-Lock connections to the PTFE tubing. The flow rate of the individual fluids was initially 100 nl/min. After the microfluidic channels were completely filled, the flow rate was reduced to 10 nl/min for the duration of the experiment. The Reynold’s number, *Re*, for the flow was 0.003 which is consistent with laminar flow conditions.

[*Re* = ρ × *v* × *d*/μ, where ρ is the fluid density, *v* is the velocity, *d* is the hydraulic diameter of the microchannel (4 × cross-sectional area/perimeter) and μ is the dynamic viscosity of the fluid].

## Supplementary information

Supporting Information.
