# Supplementary material for: Formation of pre-pore complexes of pneumolysin is accompanied by a decrease in short-range order of lipid molecules throughout vesicle bilayers
Source: Sci Rep. 2020 Mar 12;10:4585. doi: 10.1038/s41598-020-60348-0 (PMC7067851; doi:10.1038/s41598-020-60348-0)
Supplement: Supplementary file 1 — Supporting Information. [file 41598_2020_60348_MOESM1_ESM.docx]

Supporting Information

Formation of pre-pore complexes of pneumolysin is accompanied by a decrease in short-range order of lipid molecules throughout vesicle bilayers

Bayan H. A. Faraj,^a^ Liam Collard,^b,c^ Rachel Cliffe,^b^ Leanne A. Blount,^b^ Rana Lonnen,^d^ Russell Wallis,^a,e,f^ Peter W. Andrew^a^ and Andrew J. Hudson^b,f*^

**^a^ Infection, Immunity and Inflammation, University of Leicester, University Road, Leicester, LE1 9HN, United Kingdom.**

**^b^ Department of Chemistry, University of Leicester, Leicester, LE1 7RH, United Kingdom.**

**^c^ Department of Mathematics, University of Leicester, Leicester, LE1 7RH, United Kingdom.**

**^d^ Novartis Pharma AG, Basel, Switzerland.**

**^e^ Department of Molecular and Cell Biology, University of Leicester, Leicester, LE1 7RH, United Kingdom.**

**^f^ Leicester Institute of Structural and Chemical Biology, University of Leicester, Leicester, LE1 7RH, United Kingdom.**

**
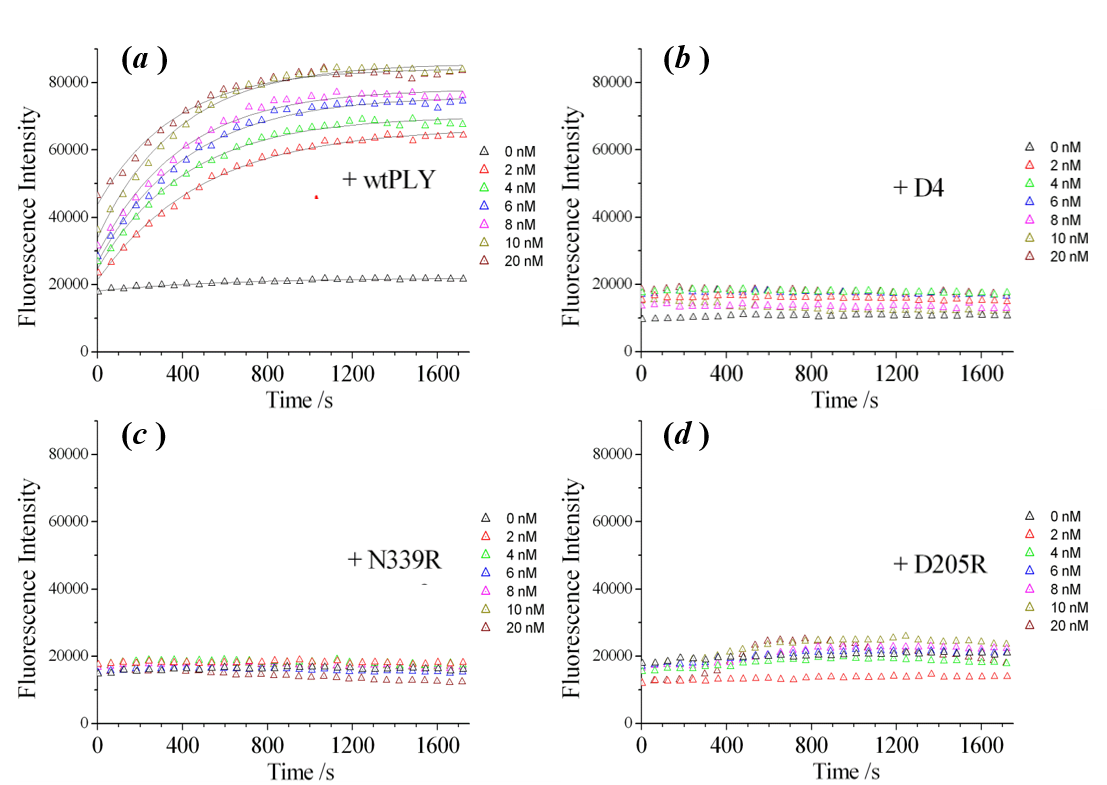
**

**Figure S1:** Leakage of calcein dye from liposomes monitored by fluorescence spectroscopy. The calcein dye was incorporated into the liposomes at a concentration that results in self quenching and a low quantum lead for fluorescence. Leakage of the dye from the interior of liposomes into the exterior solution leads to a fluorescence increase. The time course for leakage of calcein from liposome suspensions, incubated at 37 °C, was measured on a plate reader in the presences of **(*a*)** wild type (wt) PLY, **(*b*)** the truncated mutant comprising domain 4 (D4) only, and the point mutants **(*c*)** N339R, and **(*d*)** D205R at a rand of different protein concentrations.

**
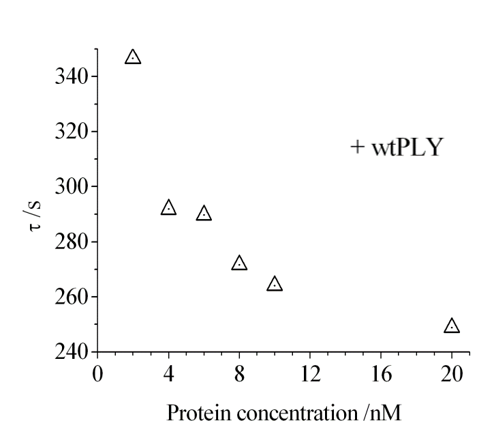
**

**Figure S2:** Rate parameters for the leakage of calcein from liposome suspensions exposed to wtPLY as a function of protein concentration. The time courses in **S1** were fitted to the function *I* (*t*) = *I*_sat._ × (1 − exp (− *t* / τ ) where *I* is the fluorescence intensity and *t* is the elapsed time. The time constant, τ , is plotted as a function of protein concentration.

**
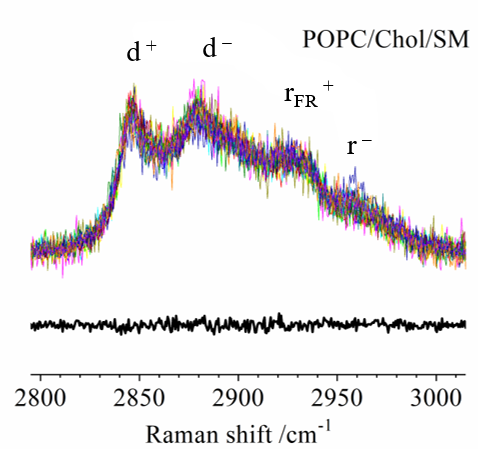
**

**Figure S3:** Comparison of Raman spectra for individually optically-trapped liposome in the absence and presence of wild-type PLY. (TOP) In the presence of PLY, the individual spectra (15 replicates) recorded from different liposomes are superimposed. (BOTTOM) Subtraction of the average spectrum recorded from 14 individual liposomes in the absence of PLY from the average spectrum recorded in the presence of PLY gives the difference spectrum. No significant features remain in the difference spectrum. Liposomes were prepared from a ternary mixture (POPC/chol/SM) with a mole ratio of 1:0.5:0.5.


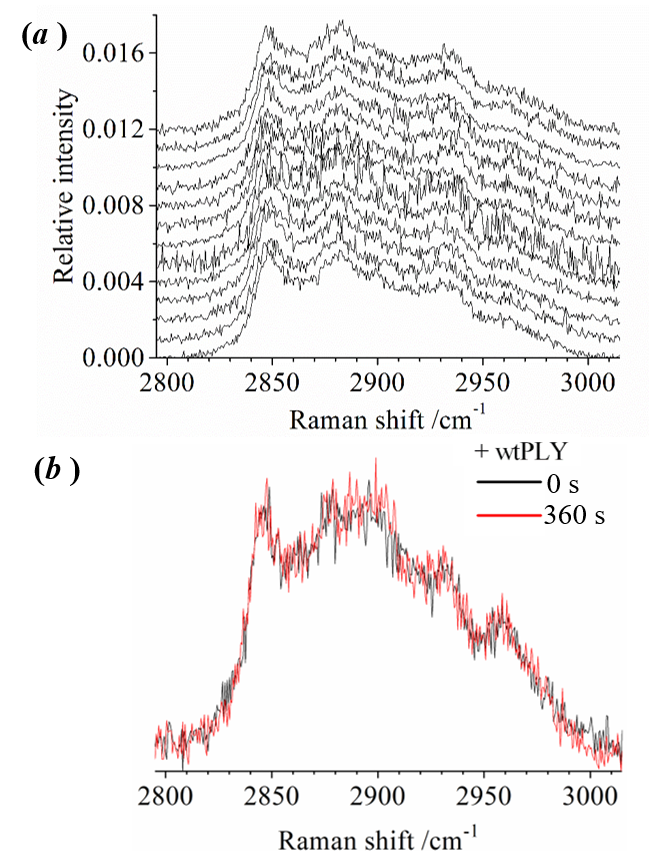


**Figure S4: (*a*)** A sequence of Raman spectra recorded at 30 s intervals from an optically-trapped POPC/Cholesterol/sphingomyelin liposome (1:1:1 mol ratio) transferred into a laminar flow of PBS buffer in the presence of PLY. **(*b*)** Comparison of the initial spectrum with the spectrum recorded after 360 s.


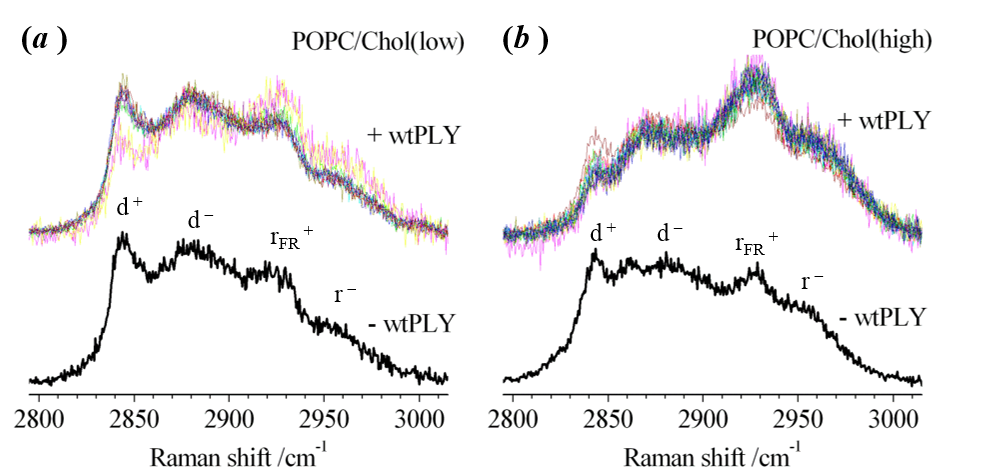


**Figure S5:** Comparison of Raman spectra for an optically-trapped liposome in the absence (-wtPLY) and presence (+wtPLY) of wild-type pneumolysin. In the absence of the protein, the spectra shown represent an average recorded from 10 different liposomes. In the presence of pneumolysin, the individual spectra recorded from different liposomes are superimposed. Liposomes were prepared from a binary mixture with a mole ratio POPC:cholesterol of **(*a*)** 4:1 and **(*b*)** 1:2. There are 10 replicates shown in both **(*a*)** and **(*b*)** for +wtPLY. In **(*a*)**, at low concentrations of cholesterol, the change in the Raman spectra is inconsistent between measurements made on different liposomes.

At high concentrations of cholesterol, in POPC/chol bilayers(1:2 mole ratio; **S5 (*b*)**), the change in the appearance of the Raman spectra is more marked than observed for equimolar amounts in **Figure 4**.As expected, the change in the Raman spectrum was more pronounced in the 1:2 POPC/chol bilayers reflecting the greater change in the membrane order, due to the presence of more cholesterol, following the phase transition from L_o_ to mixed L_o_/L_d_, which we believe to be the essential conditions for the formation of PLY oligomers (pre-pores and pores).

The 4:1 POPC/chol composition (i.e. 20 % cholesterol) for the lipid bilayer may represent the lower limit for PLY binding.^1,2,3^ In the majority of cases, there was no detectable change in the Raman measurements for liposomes (with 4:1 POPC/chol bilayers) in the presence of PLY, however, occasionally a change was detectable. Lipid bilayers with 4:1 POPC/chol will exhibit lower lipid order then 1:1 POPC/chol bilayers, and already exhibit a mixture of L_o_ and L_d_ phases (prior to PLY addition).

^1^ Huang, J., Buboltz, J. T. & Feigenson, G. W. Maximum solubility of cholesterol in phosphatidylcholine and phosphatidylethanolamine bilayers. *Biochim. Biophys. Acta - Biomembr.* **1417**, 89–100 (1999).

^2^ Ali, M. R., Kwan, H. C. & Huang, J. Assess the nature of cholesterol-lipid interactions through the chemical potential of cholesterol in phosphatidylcholine bilayers. *Proc. Natl. Acad. Sci. U. S. A.* **104**, 5372–5377 (2007).

^3^ Ibarguren, M., Alonso, A., Tenchov, B. G. & Goñi, F. M. Quantitation of cholesterol incorporation into extruded lipid bilayers. *Biochim. Biophys. Acta - Biomembr.* **1798**, 1735–1738 (2010).

**
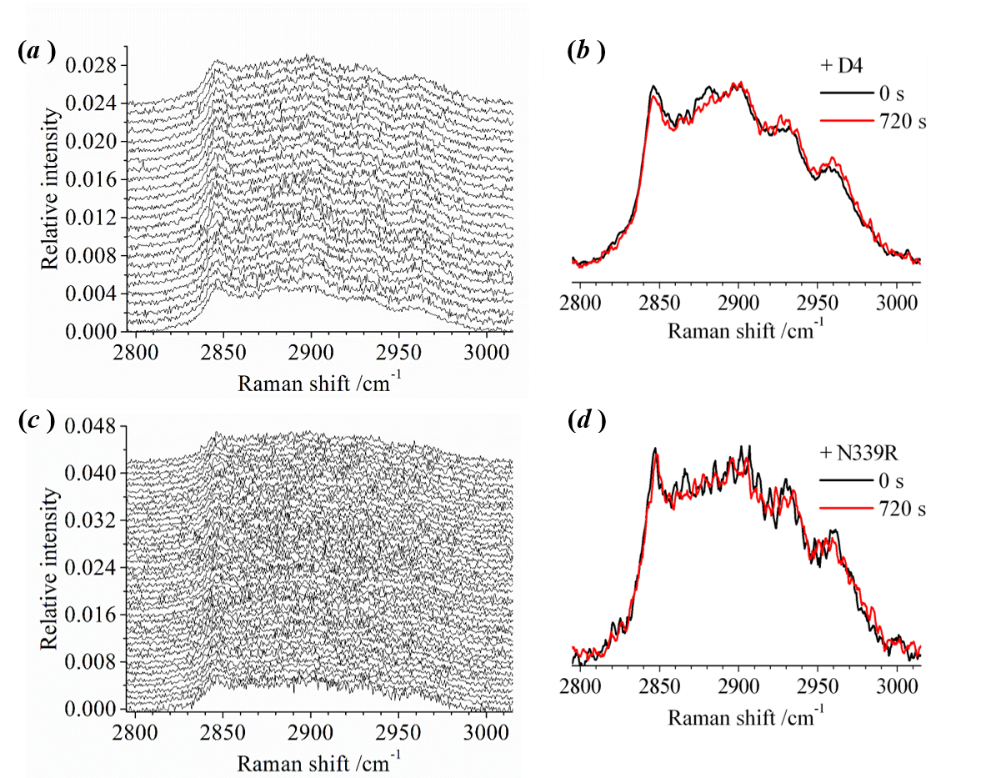
**

**Figure S6: S**equences of Raman spectra recorded at 30 s intervals from an optically-trapped POPC/Cholesterol liposome (1:1 mol ratio) transferred into a laminar flow of PBS buffer in the presence of, **(*a*)** and **(*b*)**, a truncated mutant D4 and, **(*c*)** and **(*d*)**, a point mutant N339R. [Full sequences of spectra are shown in **(*a*)** and **(*c*)**, and the initial and final spectra in **(*b*)** and **(*d*)**.]

**
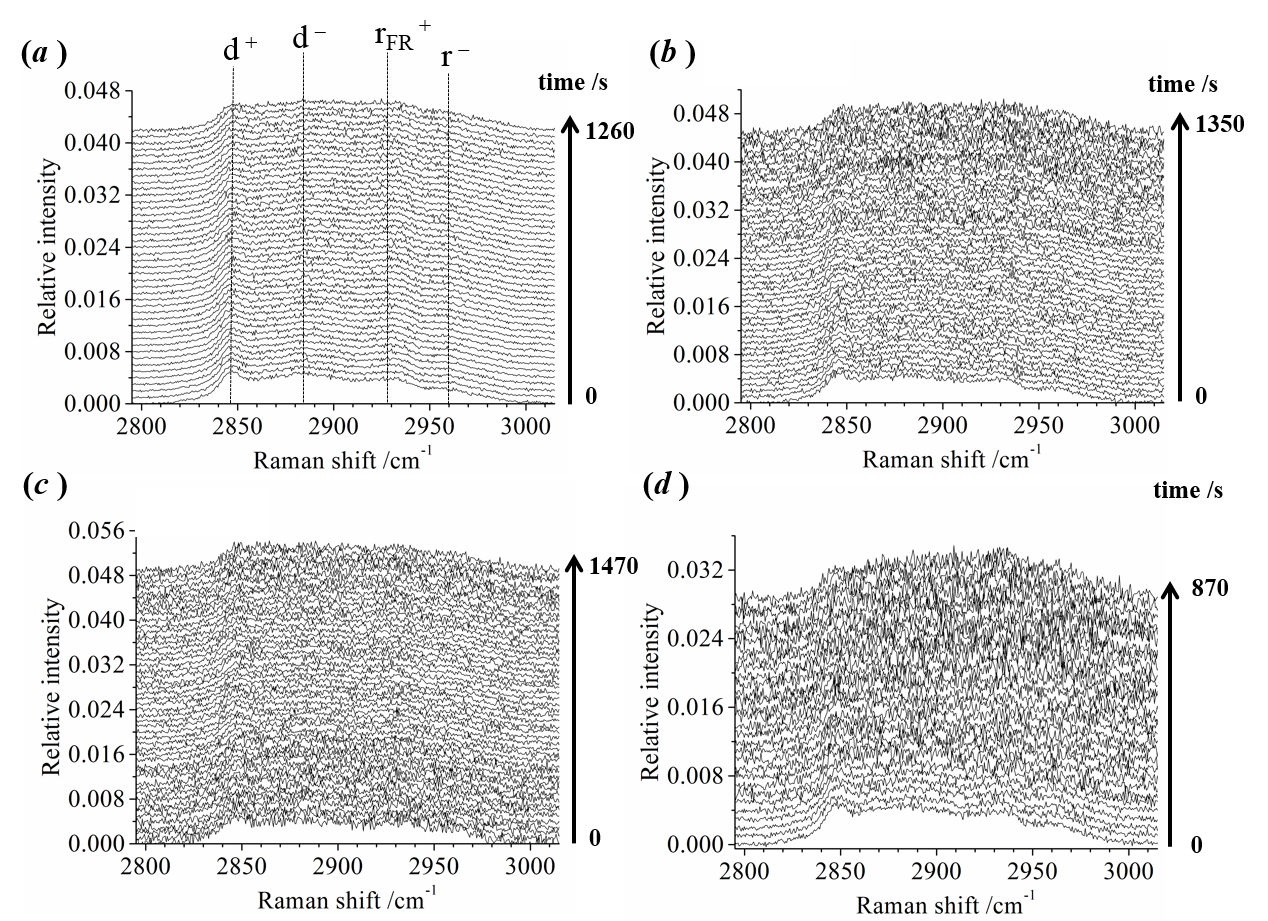
**

**Figure S7:** Experimental spectra obtained for POPC/Cholesterol liposomes (1:1 mol ratio) transferred into a laminar flow of PBS buffer in the presence of **(*a*)** D205R, **(*b*)** T55C + V163C, **(*c*)** A262C + W278C and **(*d*)** T304R. Data was analysed using MCR-ALS and the outcomes are shown in **Figure 7** and **8**.

**
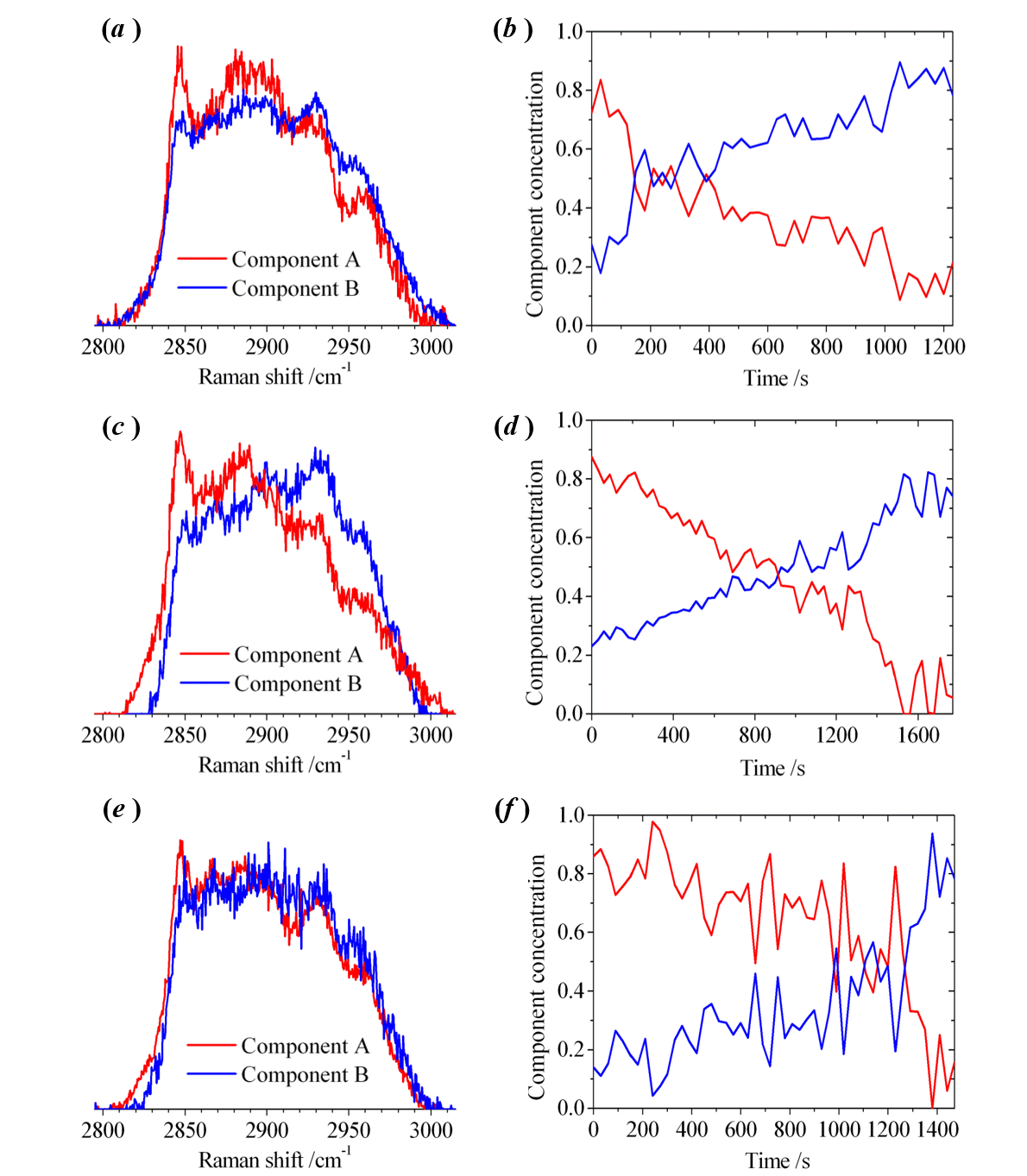
**

**Figure S8:** Multivariate-curve resolution (MCR) analysis of Raman spectra recorded from an individual optically-trapped POPC/Cholesterol liposome (1:1 mol ratio), in the region of the C-H stretching band. Sequences of spectra were recorded at 30 s intervals. The pure spectral profiles (left) and the concentration profiles (right) were obtained by MCR analysis for a liposome transferred into a laminar flow of PBS buffer in the presence of point mutants R226A [**(*a*)** and **(*b*)**], T88E [**(*c*)** and **(*d*)**] and W334F [**(*e*)** and **(*f*)**]. The results of the MCR analysis resemble those obtained for POPC/Cholesterol liposomes (1:1 mol ratio) exposed to PLY.

R226A (haemolytic activity, HA 0.06^14^) has a mutation near the perimeter of the interface between monomer subunits, and T88E (HA 0.04^14^) has a mutation located on the interior of the interface. These point mutations (both in D1) should only disrupt hydrophobic interactions with adjacent monomers by removing side chains or introducing a large charged group at the interface. W334F (HA 0.01^14^) contains a substituted tryptophan in D4 and this is expected to affect protein binding to cholesterol.^14^ D1 and D4 are understood to undergo little positional change on pore formation.^14^

**
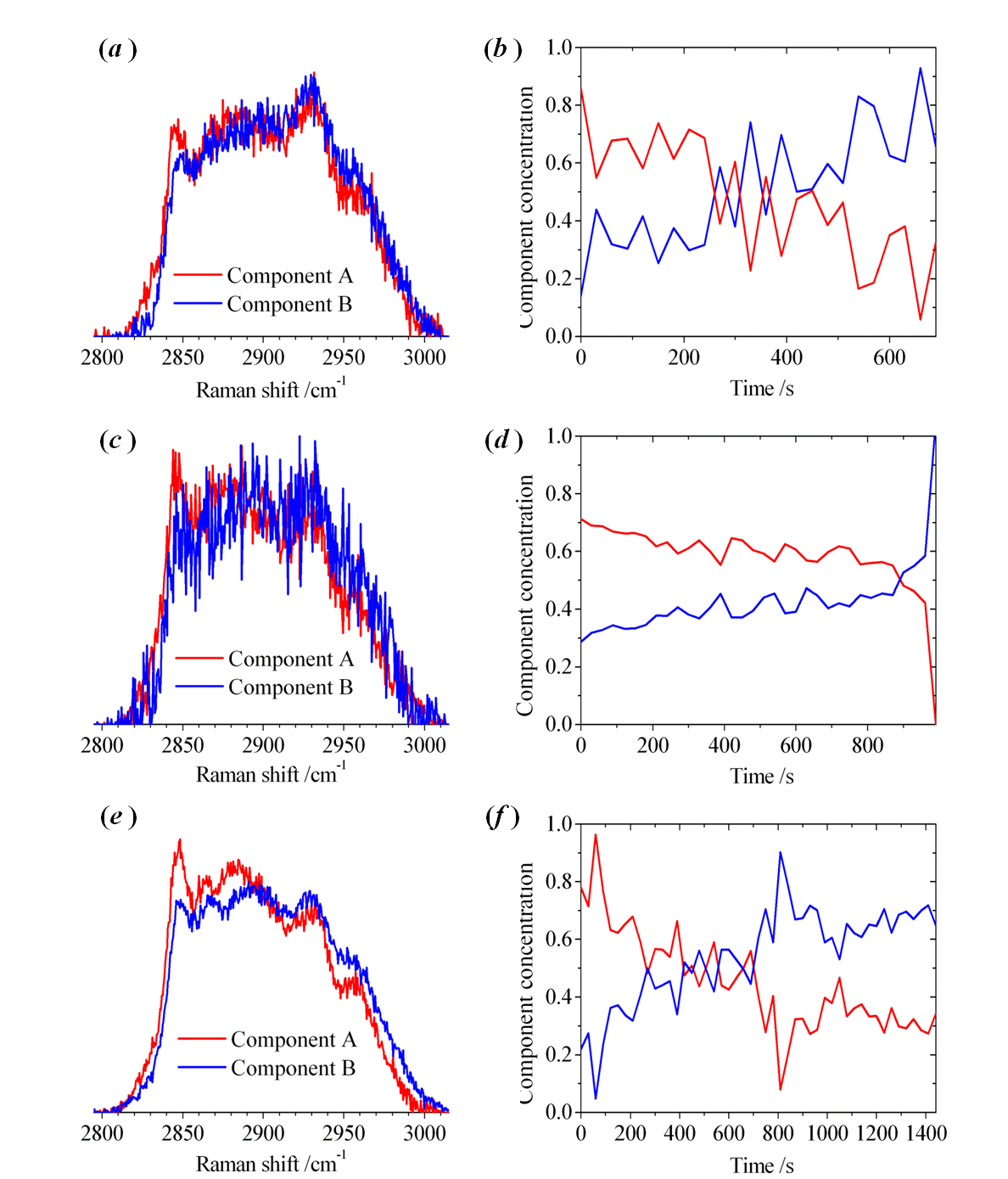
**

**Figure S9:** Multivariate-curve resolution (MCR) analysis of Raman spectra recorded from an individualy optically-trapped POPC/Cholesterol liposome (1:1 mol ratio), in the region of the C-H stretching band. Sequences of spectra were recorded at 30 s intervals. The pure spectral profiles (left) and the concentration profiles (right) were obtained by MCR analysis for a liposome transferred into a laminar flow of PBS buffer in the presence of point mutants V341R [**(*a*)** and **(*b*)**], K268A [**(*c*)** and **(*d*)**] and L11R [**(*e*)** and **(*f*)**]. The results of the MCR analysis resemble those obtained for POPC/Cholesterol liposomes (1:1 mol ratio) exposed to PLY.

V341R (haemolytic activity, HA ~1^14^) and K268A (HA 0.4^14^) have a mutation located on the interior of the interface. L11R (HA 0.7^14^) has a mutation near the perimeter of the interface between monomer subunits. These point mutations (all in D1) should only disrupt hydrophobic interactions with adjacent monomers by removing side chains or introducing a large charged group at the interface. D1 is understood to undergo little positional change on pore formation.^14^
